# Consent to Operation

**Published:** 1907-06

**Authors:** 


### CONSENT TO OPERATION.

The supreme court of Illinois has had this subject before it recently in the case of Pratt vs. Davis. The results of this trial emphasizes the necessity of surgeons having a clear understanding of their legal liabilities in understanding important operations, and the prudence of requiring explicit consent of the patient or his legal representative before beginning an operation. The decision covers three points of much interest to surgeons: 1. What is sufficient consent to an operation. 2. How much is implied in consent once given. 3. What is the privilege and duty of the surgeon in emergencies arising in the course of an operation undertaken with previously obtained consent. The case in question was as follows:

Patient married, æt. 40, had suffered from epileptic seizures for fifteen years, came with her husband to a sanitarium for treatment. There was found laceration of the womb and other conditions which required surgical interference. She was operated on for these conditions and sent home, but was not improved. She was then told to come back to the sanitarium for further operation. The surgeon then removed her womb without expressly telling her that he was going to do so, or gaining her consent, or that of her husband, though the latter was implied. The patient was no better, but rather grew worse, and later was adjudged insane and sent to the asylum. Suit for damages were instituted for removing the uterus without consent, and a verdict for $3,000 given. The supreme court affirmed the decision of the appellate court. The decision of the court on the third point is of importance, for it tends to put the duty of the surgeon in the course of an operation already undertaken in a clearer light. It is the duty and legal right of the surgeon in the presence of unexpected complications arising in the course of an operation to use his highest skill and judgment, even if the consent of the patient or his representatives cannot be obtained. It is also the right and duty of the surgeon to act in accordance with the best teachings of surgery in emergencies in which the consent cannot be obtained, even to the extent of performing operations.—Medical Herald, May, '07.

